# Two distinct *Fusarium graminearum* populations colonized European wheat in the past two decades

**DOI:** 10.1371/journal.pone.0296302

**Published:** 2023-12-28

**Authors:** Tomasz Kulik, Tomasz Molcan, Katarzyna Bilska, Marco Beyer, Matias Pasquali, Anne van Diepeningen, Kamil Myszczynski

**Affiliations:** 1 Department of Botany and Nature Protection, University of Warmia and Mazury in Olsztyn, Olsztyn, Poland; 2 Institute of Animal Reproduction and Food Research, Polish Academy of Sciences, Molecular Biology Laboratory, Olsztyn, Poland; 3 Environmental Research and Innovation Department, Luxembourg Institute of Science and Technology (LIST), Environmental Monitoring and Sensing Unit, Agro-Environmental Systems, Belvaux, Luxembourg; 4 Department of Food, Environmental and Nutritional Sciences, University of Milan, Milan, Italy; 5 Biointeractions and Plant Health, Wageningen University and Research, Wageningen, The Netherlands; Agriculture and Agri-Food Canada, CANADA

## Abstract

*Fusarium graminearum* is the main causal agent of Fusarium head blight (FHB) disease in wheat in Europe. To reveal population structure and to pinpoint genetic targets of selection we studied genomes of 96 strains of *F*. *graminearum* using population genomics. Bayesian and phylogenomic analyses indicated that the *F*. *graminearum* emergence in Europe could be linked to two independently evolving populations termed here as East European (EE) and West European (WE) population. The EE strains are primarily prevalent in Eastern Europe, but to a lesser extent also in western and southern areas. In contrast, the WE population appears to be endemic to Western Europe. Both populations evolved in response to population-specific selection forces, resulting in distinct localized adaptations that allowed them to migrate into their environmental niche. The detection of positive selection in genes with protein/zinc ion binding domains, transcription factors and in genes encoding proteins involved in transmembrane transport highlights their important role in driving evolutionary novelty that allow *F*. *graminearum* to increase adaptation to the host and/or environment. *F*. *graminearum* also maintained distinct sets of accessory genes showing population-specific conservation. Among them, genes involved in host invasion and virulence such as those encoding proteins with high homology to tannase/feruloyl esterase and genes encoding proteins with functions related to oxidation-reduction were mostly found in the WE population. Our findings shed light on genetic features related to microevolutionary divergence of *F*. *graminearum* and reveal relevant genes for further functional research aiming at better control of this pathogen.

## Introduction

*Fusarium graminearum* is a globally important pathogen causing Fusarium head blight (FHB), a devastating disease of cereals worldwide which can be caused by several *Fusarium spp*. The pathogen has biotrophic and necrotrophic (saprophytic) growth phases [1] that require adaptation to different environments like soils, plant debris, and living plants. The fungus is responsible for yield losses and contamination of the grains with mycotoxins; mainly deoxynivalenol (DON) and zearalenone (ZEA), which have important food and feed safety implications [2]. DON, which belongs to trichothecenes, inhibits the biosynthesis of nucleic acids and proteins and disrupts mitochondrial function. In addition, it negatively affects cell division and membrane integrity and induces apoptosis [3]. ZEA exhibits estrogenic activity [4]. Both mycotoxins are involved in a broad variety of toxic effects in domestic animals leading to economic losses in animal production [5,6].

*F*. *graminearum* exhibits a complex life cycle (infection, reproduction and transmission) and highly adaptive development, which is strongly affected by human activity patterns such as tillage systems, the genotypes of the crops grown, crop rotation, and fungicide use [7]. The fungus can reproduce through homothallic inbreeding, outcrossing and asexual reproduction, however, high genetic diversity and gene flow underlined that sexual reproduction remarkably shapes spatial and temporal population structures [8,9].

Emergence of *F*. *graminearum* in Europe, defined here as increasing incidence in an existing FHB population, has been documented for the first time in the Netherlands by screening a large set of isolates recovered from wheat in 2000 and 2001 [10]. Increased predominance of *F*. *graminearum* was further confirmed in other European sites [11–14].

Field isolates of *F*. *graminearum* are often studied through determination of trichothecene chemotypes/genotypes [15] to assess population diversity. Previous surveys showed that three main trichothecene chemotypes occur in Europe with predominance of 15-acetyldeoxynivalenol (15-AcDON) chemotype/genotype [16,17]. 3-acetyldeoxynivalenol (3-AcDON) appears to predominate in northern Europe [18], while nivalenol (NIV) is sporadically detected [19]. It is worth to note, however, that most of the earlier studies on *F*. *graminearum* diversity in Europe included isolates narrowed to a single geographical area. In addition, most previous population studies did not employ genomic approaches to unravel the complexity of the pathogen. Recently, one exceptional study was conducted to explore genetic diversity of a set of German isolates via restriction site associated DNA sequencing (RADseq) [8]. Genomic analyses indicated that isolates sampled from fields over a > 500 km transect belong to a single, freely recombining population displaying high degree of genetic diversity. Contrasting results could be derived from North America. Comparative genomic study by Kelly and Ward (2018) [9] detected three independently evolving and co-occurring populations of *F*. *graminearum*.

Prior to this study, we performed a phylogenomic approach incorporating a geographically diverse set of *F*. *graminearum* strains, and all known members of the *F*. *graminearum* complex [20]. We found that a phylogenomic clustering of the strains had species specific patterns within this group of cryptic species. A large *F*. *graminearum* clade contained multiple subclades with some geographic overlap, suggesting complex population structure of *F*. *graminearum* in Europe.

To address this finding, we performed phylogenomic and Bayesian analyses to gain insight into population structure of *F*. *graminearum* in Europe. The main objectives of our study were to: (1) detect and genotype SNPs of geographically diverse strains of *F*. *graminearum* at a genome-wide scale; (2) characterize the genetic diversity and population structure; (3) characterize genetic differentiation between the populations within genomic regions targeted by selection; (4) and determine gene content differences between populations. We also characterized biological functions of the genes that appeared to be population-specific and discussed the importance of our findings in terms of diversity and divergence of *F*. *graminearum*, which could be taken into account, especially in sight of future research and the development of more effective disease management.

## Materials and methods

### Fungal strains

Among the total of 96 strains used in this study, 82 were chosen to maximize geographic diversity in Europe with regard to the previous phylogenomic approach [20]. In addition, to enhance the diversity of the sample, we included twelve strains recovered outside of Europe. Two remaining strains CBS 104.09 and CBS 185.32 isolated in 1909 and 1932, respectively, were of unknown origin. Thirteen strains included into our study were isolated before 2000, when the first emergence of *F*. *graminearum* was detected in Europe [10]. Sixty-six strains were isolated from wheat, six from barley, five from soybean, two from corn, one from apple, one from giant miscanthus, one from rye and one from common vetch. For 13 strains, the host is unknown. Among the total of 96 strains analyzed in this study, eighty-two strains were recovered during the last 22 years. We also included genomic data from three other cryptic species of the *F*. *graminearum* species complex (FGSC), *F*. *boothii* (n *=* 2; CBS 110251 and CBS 119170), *F*. *gerlachii* (n *=* 2; CBS 119175 and CBS 119176) and *F*. *louisianense* (n *=* 2; CBS 127524 and CBS 127525), which served as the outgroups for phylogenetic construction. The complete detail of all strains used in this work are included in S1 Table.

### Culture conditions and DNA extraction

For DNA extraction, fungal cultures were incubated on Petri plates (Ø 80 mm) with PDA (Potato Dextrose Agar) medium at 24°C for 6 days. DNA from fungal strains was extracted from 0.1 g of mycelium with the use of the Quick-DNA Plant/Seed Miniprep Kit (Zymo Research, Irvine, CA, United States) according to the manufacturer’s protocol. DNA from each isolate was quantified on Qubit fluorometer using Qubit dsDNA BR Assay Kit (Life Technologies, USA).

### Whole-genome sequencing

Whole-Genome Sequencing (WGS) was performed as previously described in Kulik et al. 2022 [20]. Briefly, the majority of strains were sequenced by Macrogen, Inc. (Seoul, South Korea) on an Illumina HiSeq X Ten using a paired-end read length of 2 × 150 bp with an insert size of 350 bp. Libraries were prepared using the KAPA HyperPlus Kit (Roche Sequencing Solutions, Pleasanton, CA, United States). For strains: ar1 (CBS 139514), ar3 (119–12), us2 (CBS 119173), po12 (CBS 138561), po13 (CBS 138562), po14 (CBS 138563) and fbo3 (CBS 119170), whole genome libraries were prepared using a Nextera XT kit (Illumina, San Diego, CA, United States) and sequenced on the Illumina Miseq platform with the 250 bp paired-end read, version 2. Low-quality reads and adapters were removed using Trimmomatic (v.0.39) using LEADING:20 TRAILING:20 SLIDINGWINDOW:4:20 AVGQUAL:20 MINLEN:50 parameters [21]. All genomic data generated herein were deposited in the NCBI Sequence Read Archive under accession: PRJNA677929. Genome assemblies are also available for all strains analyzed in this study, and GenBank accession numbers are listed in S1 Table.

### Determination of trichothecene (TRI) genotypes

Sequence comparison of TRI12 gene belonging to the TRI core cluster enables determination of 3-AcDON, 15-AcDON and NIV genotypes [22]. To assess TRI genotypes, complete TRI12 alleles were extracted from genome assemblies of the studied strains and used for multiple sequence comparisons to reference 3-AcDON (KU572433), 15-AcDON (KU572431) and NIV (KU572430) alleles using Geneious Prime 2022.0.1. software [23]. Hits with >99.7% identity were counted as either NIV, 3-AcDON or 15-AcDON genotype. Prediction of NX-2 producers requires sequence analysis of TRI1 gene. To detect NX-2 genotypes in our set of strains, we performed sequence comparisons of extracted TRI1 alleles to NX-2 producing strain 06–204 (KM999943) [24].

### SNP calling and *de novo* assembly of unmapped reads

For SNP calling, filtered high-quality reads of 96 strains were mapped to the *F*. *graminearum* PH-1 reference genome (GCF_000240135.3) using MEM algorithms of Burrows-Wheeler Aligner (BWA) software v0.7.17 [25]. Sequence Alignment Map (SAM) files were sorted and converted to Binary Alignment Map (BAM) file with SAMtools v1.10 [26]. The average genome-wide coverage for each strain was estimated with the use of SAMtools. The mapped reads were used to call SNP variants with the Genome Analysis Toolkit (GATK) v4.2.0.0. [27]. First, SNPs from each strain were determined by HaplotypeCaller with the options: -ERC GVCF—minimum-mapping-quality 20—sample-ploidy 1. Afterward, the GenomicsDBImport tool was used to combine a single GVCF file into one and following joint variant calling was performed by GenotypeGVCFs using the option:—max-alternate-alleles 4. The raw VCF file was filtered by VariantFiltration tools, using a function to hard filter SNPs with quality thresholds recommended by GATK. The following thresholds were used: QUAL < 30, QD < 2.0, SOR > 3.0, FS > 60.0, MQ < 40.0, MQRankSum < -12.5 and ReadPosRankSum < -8.0. Additionally, a second round of filtration was performed by means of VCFtools v0.1.17, using the following parameters:—maf 0.05—max-missing 0.80—minQ 30. Final analysis included evaluation of single nucleotide polymorphism (SNP) variants. To decipher unmapped regions that were absent in reference PH-1 strain (< 80% identity over > 50% of the read), we also performed de novo assembly of unmapped reads using SPAdes (v.3.13.2) [28] with k-mer values of 21, 33, 55, 77, 99, 127 and using the “careful” option to reduce mismatches. We applied filters to remove regions with less than 5X coverage per genome. Augustus version 3.2 [29] was used to employ a Hidden Markov model to predict genes on these contigs, utilizing validated parameters settings based on experimentally validated introns from *F*. *graminearum*. Coding sequences were found on both strands of DNA, involving ATG start and stop codons. Orphan contigs represent contigs constructed from unmapped reads, while orphan genes are genes annotated on orphan contigs.

### Population genomic structure

Population analysis required as an input independent SNPs i.e. with no correlation between them. Therefore, linkage disequilibrium (LD) pruned SNPs with minor allele frequency (MAF) > 0.05 were used. LD-pruned datasets were obtained using PLINK v1.9 software [30] with the option:—indep-pairwise 50 10 0.1. The input set after filtering and LD pruning resulted in 9055 SNPs. Population structure was analyzed using the model-based Bayesian analysis implemented in STRUCTURE [31]. The number of subpopulations (K) was determined using the mean likelihood values in the ΔK method and the lnP (K) values [32,33] calculated by Structure Harvester [34]. We estimated the variance between replicates by continuously running K  =  1–8 to determine the optimal population number [35]. The analysis was conducted with a burn-in of 600,000 iterations followed by 1,400,000 Markov Chain Monte Carlo (MCMC) replications in seven independent runs. No previous information was used to define the clusters. To evaluate the clustering findings, we forced K to its true value. For each given K value, the run with the highest likelihood was used to cluster the accessions. We set the threshold value at 0.8 to distinguish between the pure and mixed groups [36].

The Principal Components Analysis (PCA) was also used to determine *F*. *graminearum* population structure. PCA was calculated by PLINK v1.9 software with following parameters:—make-bed—pca—maf 0.05—geno 0.2. Results were visualized with the use of ggplot2 package v3.4.2 implemented in R software v4.3.0. Phylogenomic analysis was conducted to estimate genetic relationships among studied strains. The VCF file was converted to PHYLIP format using vcf2phylip tool v2.8 [37]. Phylogeny was estimated using maximum-likelihood (ML) inference in IQTree v2.0.6 [38], using the TVM + F + R5 substitution model with 1000 bootstrap replicates. Two strains *F*. *boothii* (CBS 110251 and CBS 119170), two *F*. *gerlachii* (CBS 119175 and CBS 119176) and two *F*. *louisianense* (CBS 127524 and CBS 127525) were used as an outgroup.

To detect differences in clustering between populations, we compared the trees with and without outliers using the “cophylo” function in Phytools [39]. SplitsTree v4.19.0 [40] was used to create a distance-based split network using the neighbour-net algorithm. In population genomic research, the frequency of genetic recombination can be studied by Linkage Disequilibrium (LD) decay [41. LD half-decay distance is usually used to predict sexual frequency of fungi, which varies from 110 bp for outcrossing *Schizophyllum commune* to >100kb for highly clonal, *Batrachochytrium dendrobatidis* and *Candida albicans* [41]. Linkage disequilibrium decay analysis was calculated by means of PLINK 1.9 software using a sliding window of width 10kb. Next, the mean values of LD across 10 kb sliding windows from any SNPs was calculated. Smoothing curves were fitted to the mean LD decay values using R v4.3.0 build function *loess*. To determine the approximate distance required to reach LD_**50**_ decay value, the closest point above 50% of maximum linkage from start position was estimated. In the scenario that one of the populations had a significantly higher number of SNPs that the other we would have a finer resolution to detect recombination events. To address this scenario, we used the identical number of SNPs (50,000) randomly sampled along the genome from both EE and WE populations for the LD analysis.

### Detection of genomic signatures of selection

Several population genetic summary statistics were generated using non-overlapping 10 kbp sliding windows and the PopGenome R package [42] to determine whether regions in the EE and WE populations were affected by selection. Linkage disequilibrium analysis in the EE and WE populations, which become negligible at distances greater than 10 kb, was used to determine the window size. The within-population diversity was assessed by calculating π (nucleotide diversity, the average number of differences between individuals) [43], θ (nucleotide polymorphism based on segregating sites) [44] and Tajima’s D [45] and estimated the number of recombination events within a population using R_M_ (four-gamete test) [46]. The differentiation between populations were quantified by assessing pairwise values of D_xy_ (nucleotide divergence based on the absolute number of differences between two populations) [43,47], F_ST_ (per haplotype, adjusted for unequal population sizes using weighted averages) [48], and Tajima’s D (estimated as a relative measure of inter-population divergence for pooled populations) [49]. Genome-wide averages of summary statistics were compared among both populations using R statistical software and visualized using Circos [50]. A nonparametric permutation approach for genome-wide SNPs has been adjusted to assess the relevance of 10kb sliding-window statistics with regard to the genomic distribution of values for each population as described by Kelly and Ward (2018) [9]. A customized shell script and BedTools were used to generate the set of 1000 random variations of the VCF file [51]. The VCF file carried chromosomal coordinates of each SNP and the associated genotypes belonging to both EE and WE populations. Each permutation generated a random set of SNP coordinate/genotype combinations that maintained the data’s joint site-frequency spectrum but were unrestricted by linkage disequilibrium (i.e., spatially randomized). This involved randomizing observed genotypes across the SNP coordinates. In order to produce a null genome-wide distribution of summary statistics for each population and all pairwise combinations of populations, the sliding-window technique was then applied to each of the 1000 permuted datasets. At P-value 0.05, or the probability that the observed value was more extreme than values from the null distribution, observed values for a 10 kb window were deemed outliers. As a result, windows that displayed localized signals of linkage disequilibrium linked to selective sweeps and had patterns of variation that were significant in the context of each population’s genome-wide diversity were referred to as outliers. Genomic regions of interest were restricted to those with significant absolute and relative differentiation between the two populations [49] (significant FST, Dxy, and Tajima’s D in at least two population comparisons), along with significant reductions in nucleotide diversity (π), in order to lower the likelihood of identifying false positives.

### Quantifying differences in gene content

To assess the differences in gene content among strains, coding sequences were recovered from reads that corresponded to putative genes on orphan contigs. Additionally, annotated PH-1 protein-coding sequences were included as an internal reference. First, orthologous gene sequences were identified with the use of the micropan R package [52]. All pairwise BLAST comparisons were conducted on predicted protein sequences using blast+ software (v2.13.0) [53]. The BLAST alignment scores (bitscore) were used to calculate pairwise distance values (D_ij_). The protein sequences were grouped into orthologous groups using single linkage clustering with *D*_*i*,*j*_ ≥ 0.5 threshold. Next, a pan-genome matrix including the number of copies of each ortholog per genome was then constructed based on sequence clustering. Genes encoding proteins that were present in the reference genome and all other genomes were categorized as core genes, while protein encoding genes that were missing in one or more genomes were termed as accessory genes. To identify differentially conserved genes across populations, the gene enrichment test (E test) adopted from den Bakker et al. (2011) [54] was used to compare the relative frequency of each accessory gene in two *F*. *graminearum* populations. Genes were considered differentially conserved when the test statistic (E-test) exceeded two standard deviations of the population mean, i.e., an empirical P-value < 0.05.

### Functional annotation and enrichment analyses

The annotation of differentially conserved genes was performed by eggnog-mapper (v2.1.10) [55]. Putative functions and homologues of these proteins were further explored against fungal database using blast+ software (v2.13.0) [53]. Interpro analysis was also performed using InterPro Scan [56] to search for InterPro domains, GO terms, and protein family relationships.

## Results

### Population genomic structure of *F*. *graminearum*

We generated whole-genome sequences for 96 strains of *F*. *graminearum* and three other cryptic species of the FGSC, *F*. *boothii* (n *=* 2), *F*. *gerlachii* (n = 2) and *F*. *louisianense* (n *=* 2). On average, genome sequencing of the *F*. *graminearum* strains resulted in 68X coverage of the PH-1 reference genome (S1 Table). Based on the mapping results, we detected 705,519 SNPs among all *F*. *graminearum* strains. The number of SNPs increased to 1,440,145 after including *F*. *boothii*, *F*. *gerlachii* and *F*. *louisianense*. The number of high-quality SNPs retained after filtration decreased to 240,169 within *F*. *graminearum* and to 286,508, when including strains from other species of FGSC. Firstly, we used PCA and STRUCTURE to analyze the genetic structure of *F*. *graminearum*. Both analyses were performed using 9055 LD-pruned SNPs (MAF > 0.05). Results of the STRUCTURE analysis using the Delta K method, showed that the Delta K had the maximum value at K  =  3 (S1 Fig).

**Fig 1:**
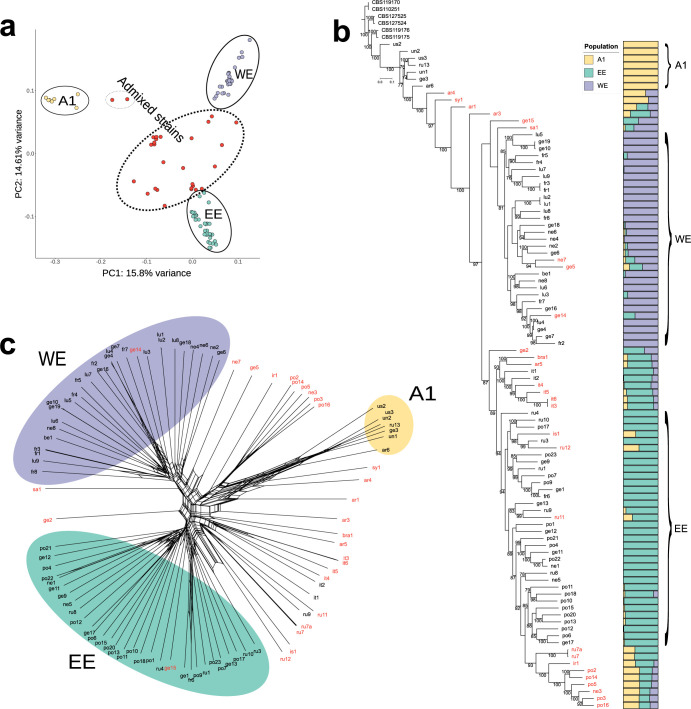
Population structure analysis of *F*. *graminearum* populations. (a) Principal component analysis (PCA) of a set of *F*. *graminearum* strains detecting three distinct populations: A1, East European (EE) and West European (WE). Legend: A1 includes seven strains of diverse geographical origin. (b) Evolutionary history and genetic structure of European populations of *F*. *graminearum*. Legend: A maximum-likelihood (ML) phylogeny inferred from SNPs identified by reference-based mapping of whole genome sequences to the reference PH-1 strain (accession number GCF_000240135.3). Three different colors indicate clustering assignment of each isolate in the three populations inferred from Bayesian analyses: A1 = yellow, East European (EE) = green and West European (WE) = purple. Strains marked with red font indicate admixed strains. Six strains from FGSC were chosen as outgroup: *F*. *boothii* (CBS 110251 and CBS 119170), and *F*. *louisianense* (CBS 127524 and CBS 127525) and *F*. *gerlachii* (CBS 119175 and CBS 119176). The tree was rooted with *F*. *boothii* and drawn to scale, with branch lengths measured in the number of substitutions per site. (c) A phylogenetic network constructed from a set of *F*. *graminearum* strains by reference-based mapping to the reference PH-1 strain. Legend: Strains are represented by terminal nodes, and relationships are depicted as branches with parallel edges indicating recombination and/or gene transfer. Strains marked with red font indicate admixed strains.

*F*. *graminearum* populations have been referred to as: A1, EE (East European), and WE (West European). The names of the two large populations (EE and WE) were assigned according to the geographic origin of the majority of the strains within them. The WE population included a set of twenty-eight strains of West European origin (France, Germany, Luxembourg and the Netherlands). Thirty-three strains were grouped into the EE population, among them the majority (n = 22) were from Eastern Europe (mostly Poland and Russia). Among them, five strains (ru1, ru3, ru4 and ru10) were recovered from different Russian regions of continental border between Europe and Asia. The A1 population included only seven strains of diverse geographical origin. The detailed list of strains assigned to the defined populations is provided in S1A Table. PCA confirmed three main clusters that corresponded to the results obtained with STRUCTURE. The first two principal components accounted for 30.41% of genetic variation in the data (Fig 1A).

In the phylogenomic tree, strains belonging to WE and EE populations were clustered into two well supported (97% of bootstraps) sister clades (Fig 1B). Strains belonging to the A1 population were placed close to the root of *F*. *graminearum* lineage on the tree. STRUCTURE indicated that twenty-eight admixed strains exhibited different proportions of ancestry with *F*. *graminearum* populations. They were dispersed between three populations on the PCA (Fig 1A) and phylogenetic network (Fig 1C). In the phylogenomic tree (Fig 1B), most of the admixed isolates occupied basal positions within population-specific clades, consistent with expectations of their recombinant background. Other admixed strains (ru7, ru7a, ir1, po2, po14, po5, ne3, po3 and po16) were grouped into separate clusters with high bootstrap value. We found, however, that six admixed strains (is1, ru12, ru11, ne7, ge5 and ge14) were placed into either EE or WE cluster in the ML tree.

**Fig 2:**
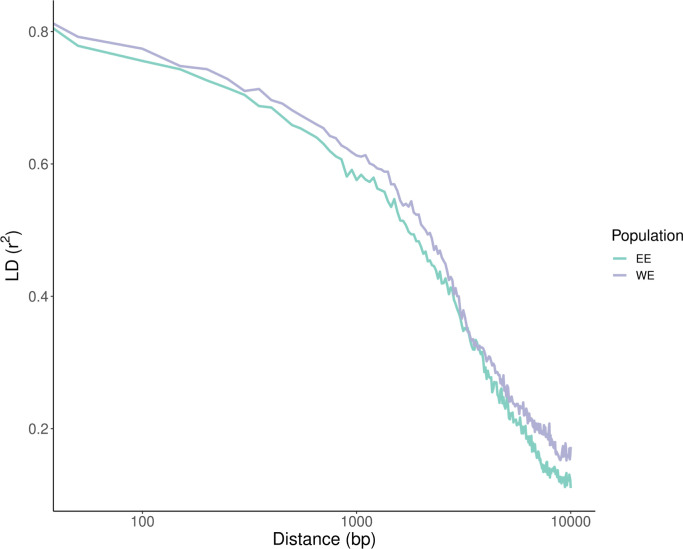
Linkage disequilibrium (LD) decay between pairs of SNPs, measured as R2, with distance on the same scaffold.

Some inconsistency in the determination of admixture could be also observed when comparing results from PCA and phylogenetic network. For example, ge15 and ge14 were clustered into defined populations on the phylogenetic network, but STRUCTURE and PCA indicated their admixture history. We excluded all admixed strains (n = 28) from further population genetic analyses. In addition, A1 population was excluded from further analysis due to the small sample size. The decrease in LD (R2) with physical distance for the two populations is shown on Fig 2. For both EE and WE populations, maximum LD was 1. Minimum LD for population EE was 0.000157 and for population WE, it was 0.0017. Both WE and EE populations showed similar LD_50_ values, which were 1783 and 1991 for EE and WE, respectively.

### Determination of trichothecene (TRI) genotypes

**Fig 3:**
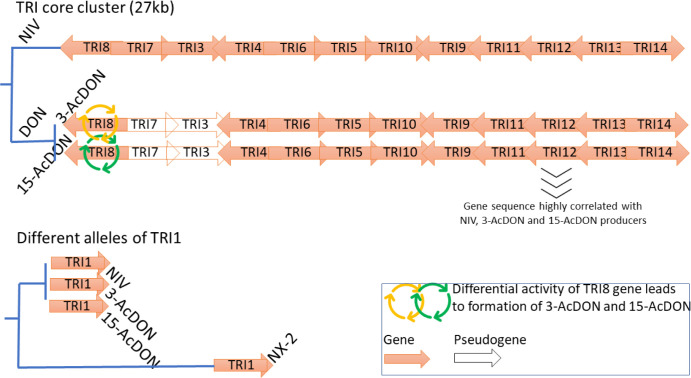
Genetic variation of TRI genes leading to formation of NIV, 3-AcDON, 15-AcDON and NX-2 genotypes.

TRI12 alleles from each studied strain were compared to reference alleles of 3-AcDON, 15-AcDON and NIV genotypes to determine trichothecene variation within the studied set of strains. Results of TRI-genotyping are shown in S1 Table. The variation in the distribution of TRI genotypes varied among *F*. *graminearum* populations, being more prominent in EE than WE population. In the EE group, 76% (n = 25) of strains were identified as 15-AcDON genotype, 8% (n = 6) as 3-AcDON and only 6% (n = 2) as NIV genotype. All strains belonging to the WE population were determined as 15-AcDON genotypes. NX-2 genotype is determined in cytochrome P450 monooxygenase encoded by TRI1 (Fig 3). To detect NX-2 genotypes in our set of strains, we performed sequence analysis of the TRI1 alleles of studied strains against NX-2 producing strain 06–204 (KM999943) [24]. Results of the analysis showed a 1.9–2.2% difference between the NX-2 producer and the TRI1 alleles from other strains, which indicates that the TRI allele typical for NX-2 producers is absent in European populations of *F*. *graminearum*.

### Genome-wide divergence between *F*. *graminearum* populations

Non-overlapping, 10 kb sliding window analysis revealed that both EE and WE populations exhibited only slightly different levels of diversity in terms of average nucleotide diversity (π) and polymorphism (θ) (Table 1). The genome average of positive Tajima’s *D* values reported for both populations does not indicate population expansion but suggests evidence for balancing selection and/or population bottlenecks. In addition, positive average Tajima’s *D* and large number of pairwise nucleotide differences suggest that both populations display increased heterogeneous nature, presumably resulting from recombination. The F_ST_ is a measure of population differentiation due to genetic structure. An F_ST_ value greater than 0.15 can be considered as significant in differentiating populations [57,58]. We found that differentiation between two populations was significant with an average F_ST_ = 0.278 and the mean divergence D_xy_ = 0.0016277%.

**Table 1:** Average genomic diversity within *F*. *graminearum* populations.

Population	θ^1^	π	Tajima’s D
EE (East European)	0.001003	0.001264 ^a^	0.772
WE (West European)	0.000978	0.001218 ^b^	0.804

^1^ nucleotide polymorphism per site based on the number of segregating sites.

(^a^-^b^) Nonparametric tests were used to compare the genomic distributions of each sliding-window summary statistic among populations. Different letters indicate significant (P-value < 0.001) differences in the population distributions.

### Genomic regions involved in population divergence

**Fig 4:**
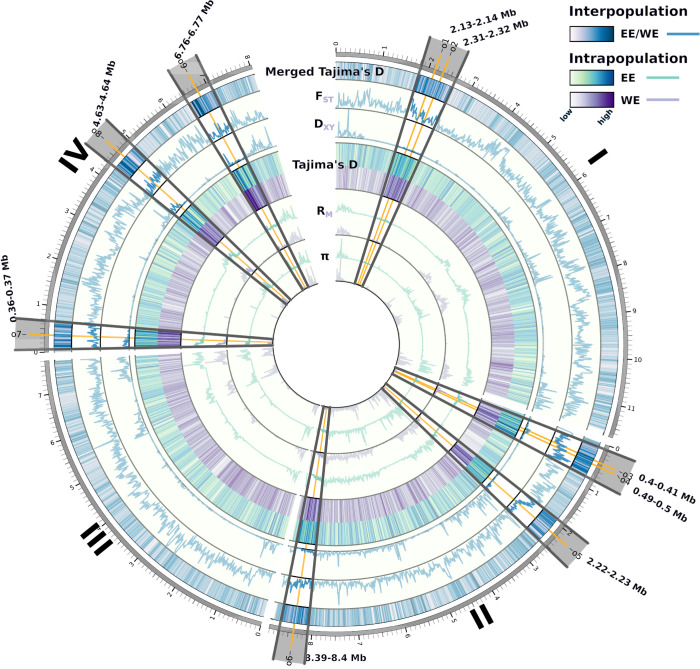
Distribution of outliers with signatures of selection. Legend: Sliding-window values of interpopulation differentiation (Panel B, Tajima’s D (estimated as a relative measure of inter-population divergence for pooled populations), D_xy_ (nucleotide divergence based on the absolute number of differences between two populations), and F_ST_ (per haplotype, adjusted for unequal population sizes using weighted averages) were calculated in 10 kb windows to identify outliers in both East European (EE) and West European (WE) populations. The nine outliers (o1-o9) showed significant (*P-value* < 0.05) divergence between populations based on pairwise (interpopulation) values of Tajima’s D, D_xy_, and F_ST_, coupled with significantly reduced diversity (π) within the populations. Significance was assessed by comparing observed sliding-window values of each metric against a null genome-wide distribution derived through random permutation.

We identified eleven regions with genetic signatures of selection (i.e., outliers) (S1 Table, Table 2, Fig 4) based on statistics that were collected from segments identified by a sliding window over *F*. *graminearum* genomes. These outliers exhibited significant interpopulation divergence (*P-value <* 0.005 for *D*_*xy*_, *F*_*ST*_ and Tajima’s *D* between populations) and reduced diversity (*P-value <* 0.05 for *π*) within at least one population by comparing to a null distribution of values produced from random permutation. We also confirmed that excluding outliers from phylogenomic approaches had no impact on topology of phylogenetic network (S2 Fig) and did not affect clustering of the strains into defined populations on the phylogenomic tree (S3 Fig). Correspondingly, the results inferred from STRUCTURE and PCA with or without outlier regions were similar (S2 Fig).

**Table 2:** List of genes found in outliers linked to pathogenicity/virulence and fungicide resistance of pathogenic fungi.

Protein ID	Family relationships	Justification of selection	Reference (s)
SCB64107.1	Amidase (IPR000120)	Analysis of a hypovirulent mutant of *Sclerotinia sclerotiorum* revealed that a mutation in an amidase-encoding gene, Sscle_10g079050, resulted in reduced virulence, likely through affecting oxalic acid homeostasis.	[59]
SCB65560.1	Mg2+ transporter protein, CorA-like/Zinc transport protein ZntB (IPR002523)	*Verticillium dahliae hex1* mutant exhibited diminished ability to colonize and cause disease on eggplant.	[60]
		Deletion of *hexA* gene in *Aspergillus flavus* reduced the production of asexual spores and decreased virulence on peanuts and maize seeds.	[61]
		Deletion of *hexA* gene in *Magnaporthe grisea* reduced the pathogenicity and survival during nitrogen starvation.	[62]
		Deletion of *hexA* gene in *F*. *graminearum* reduced the production of asexual spores and reduced virulence on wheat spikelets.	[63]
CEF77069.1	Major facilitator superfamily (IPR011701)	*Alternaria alternata* mutant lacking *AaMFS54* produced fewer conidia and increased sensitivity to many potent oxidants (potassium superoxide and singlet-oxygen generating compounds), xenobiotics (2,3,5-triiodobenzoic acid and 2-chloro-5-hydroxypyridine), and fungicides (clotrimazole, fludioxonil, vinclozolin, and iprodione).	[64]
CEF85567.1	Synthase CPS1 (CEF85567)	Deletion of the cps1 in *Botrytis cinerea* perturbs hyphal expansion after 24 h and resulted in a severe reduction of mycelial growth in a solid medium and modified hyphal aggregation into pellets in liquid cultures. It strongly impairs plant penetration, plant colonization and the formation of sclerotia.	[65]
CEF79590.1	Carbonic anhydrase (IPR001765)	Carbonic anhydrase is involved in CO2 sensing and virulence of *Candida albicans* and *Cryptococcus neoformans*.	[66]

In seven outliers, evidence of selection was limited to a single population (5 for WE and 2 for EE population), implying that a single selective sweep had targeted the individual population. In the remaining two outliers both EE and WE populations exhibited evidence of selection, which is indicative of soft sweeps, where more than one haplotype has swept through the population [31,67].

Proteins found in outlier regions were further analyzed using InterProScan [56] to identify functional protein domains, assign Gene Ontology (GO) terms and predict protein families (S1 Table). Of the 38 proteins, 26 were annotated with functional domains or overlapped with predicted gene families. Twenty-one proteins were associated with matching Gene Ontology (GO) terms. The most common “GO terms” were: protein binding (GO:0005515) (n = 7), zinc ion binding domains (GO:0008270) (n = 4), regulation of DNA-templated transcription (GO:0006355) (n = 3), transmembrane transport (GO:0055085) (n = 3), DNA-binding transcription factor activity, RNA polymerase II-specific (GO:0000981) (n = 2) and membrane (GO:0016020) (n = 2). Additionally, we searched the literature for studies showing involvement of the detected proteins in pathogenicity and virulence and fungicide resistance of fungal pathogens (Table 2). We selected four proteins (SCB64107.1, SCB65560.1, CEF85567.1, CEF79590.1), previously reported in the literature to be associated with pathogenicity/virulence in different pathogenic fungi. One protein (major facilitator superfamily, CEF77069.1) has been previously linked to resistance to different xenobiotics including fungicides [68]. Three proteins (SCB65560.1, CEF77069.1, CEF85567.1) were also linked to spore production, which is critical to the dispersal of pathogens in the fields [69].

### Gene content difference between EE and WE populations of *F*. *graminearum*

We performed a de novo assembly of the unmapped reads from each strain in order to reveal accessory genes that might display population-specific conservation. Among the total of 26924 identified accessory proteins, 23 were differentially conserved among populations (E test P-value < 0.05). Among them, sixteen were functionally characterized based on InterProScan analysis (S1 Table). Most accessory proteins (n = 21) were completely absent or present in a single strain from the EE population but were found in the majority of WE strains. Further BLAST analysis showed that most of them had >70% identity (q-cover >90, E-value = 0) to proteins from species outside FGSC or even in some cases to fungi other than fusaria (S1 Table).

One protein (EYB25392.1) uniquely conserved in the WE population was assigned to polyketide synthase (PKS), which belongs to a large, multidomain enzyme family that synthesizes a wide range of secondary metabolites [70]. Among proteins associated with matching Gene Ontology (GO) terms three proteins were associated with transcriptional and translational regulations: regulation of DNA-templated transcription (GO:0006355), DNA-binding transcription factor activity, RNA polymerase II-specific (GO:0000981), nucleotide binding (GO:0000166), tRNA aminoacylation for protein translation (GO:0006418), tRNA aminoacylation for protein translation (GO:0006418). Other proteins were associated with protein binding (GO:0005515) (n = 2), zinc ion binding domains (GO:0008270) (n = 2), iron ion binding (GO:0005506) (n = 2) and membrane (GO:0016020) (n = 2). Four other proteins were associated with functions related to oxygen-dependent metabolisms: oxidoreductase activity (GO:0016491), oxidoreductase activity, acting on paired donors, with incorporation or reduction of molecular oxygen (GO:0016705), monooxygenase activity (GO:0004497).

## Discussion

Phylogenomic analyses of geographically diverse strains of *F*. *graminearum* provide evidence for the existence of two fungal populations in Europe. Both of them exhibit considerable levels of genetic diversity and substantial gene flow within and among populations, which, however, did not disrupt genetic differentiation between these populations. The evidence of recombination in *F*. *graminearum* in Europe has been previously documented on different German field isolates [8]. However, despite evidence of their high degree of genetic diversity, previous surveys did not support genetically distinct populations, likely due to restricted sampling period/areas that might not allow the appearance of a genetic structuring. We found that *F*. *graminearum* populations display various spatial distribution patterns in Europe. The EE population is primarily prevalent in Eastern Europe, but EE strains can be also found in western (Germany, France and Netherlands) and southern areas (Italy). In contrast, the WE population appears to be endemic to Western Europe. The majority of the strains in both groups was isolated from wheat (26 out of 28 strains from WE and 22 out of 33 strains from EE) with the association between host and geographic population being non-significant (Chi-square test, P>0.05), suggesting that the species of the host plant did not play a significant role for the differences observed between EE and WE.

Our study also identified a small A1 population, which consistently with our previous findings [20], was placed close to the root of the *F*. *graminearum* lineage on the phylogenomic tree (Fig 1B). A placement at the basal position of the tree indicates its close genetic relationship to the ancestor of European populations. Previous phylogenomic analysis showed a close genetic relationship of A1 strains to PH-1 reference strain [20], recovered in late 1990s in Michigan [71]. We hypothesize that A1 population arises from NA1 population historically predominating in North America [72]. NA1 might have been introduced to Europe in the past and had vanished through population replacement, as only one A1 strain (CS10007/ge3) of European origin recovered over 15 years ago has been detected in this study. In addition, the detection of a considerable number of admixed strains with A1 background (Fig 1B) suggests that frequent backcrossing might have highly reduced its size in the past. The LD decay patterns revealed in this study could be compared to heterothallic ascomycetous yeast *Lachancea kluyveri* and *Saccharomyces cerevisiae* (Wine/European) [41] suggesting a mixed reproductive mode with clonality and rather occasional sexual reproduction of studied strains. Occurrence of recombination within populations is consistent with the shape of the SplitsTree diagram (Fig 1C), where both, EE and WE were characterized by a complex network with many reticulations.

Emergence of *F*. *graminearum* in Europe, has been documented for the first time in the Netherlands in 2000 and 2001 growing seasons [10] and further confirmed in other European sites including western, southern and eastern areas [13,14]. Most of the strains assigned in our study to either the EE or WE population were recovered after 2000, which suggests strikingly that *F*. *graminearum* emergence in Europe could be linked to two independently evolving populations. In this study, we indicated that their approximately simultaneous emergence resulted from population-specific selection pressures that led to the formation of unique localized adaptations enabling them to migrate in their environmental niche. We demonstrated that the divergence of populations mainly results from selective events that differentially targeted both of them.

*F*. *graminearum* field isolates are often characterized through determination of trichothecene chemotypes/genotypes, which allows tracking changes in field populations over time [19,73,74]. Recently, Kelly and Ward (2018) [9] demonstrated that genetic populations of *F*. *graminearum* in North America display unique patterns of genetic variation in genes responsible for trichothecene production. For example, a recently emerged population NA2 includes 3-AcDON genotypes, while the other NX-2 population contains isolates producing novel NX-2 mycotoxin. 15-AcDON genotypes are primarily found in the NA1 population [75]. We demonstrated that strains with the 15-AcDON genotype dominated both EE and WE (as well as A1) populations. Predominance of 15-AcDON is consistent with previous surveys showing high incidence of this genotype among field isolates sampled from different European sites [16]. However, there are differences in the distribution of other genotypes among two populations. While the 3-AcDON and NIV genotypes are present in the EE group, the 15-AcDON genotype is solely found in the WE population. The apparent lack of clear TRI genotype-related differences between populations confirms our results of genome scans, which did not uncover signatures of selection within the TRI core cluster (S1 Table).

Dynamic interactions between hosts and pathogens result in positive selection on proteins responsible for pathogenesis/virulence. In this study, we identified four proteins with signatures of selection (SCB64107.1, SCB65560.1, CEF85567.1, CEF79590.1), which have been shown to contribute to pathogenicity/virulence in different fungal pathogens such as: *Sclerotinia sclerotiorum* [59], *Verticillium dahliae* [60], *Aspergillus flavus* [61], *Magnaporthe grisea* [62], *Botrytis cinerea* [65], *Candida albicans* and *Cryptococcus neoformans* [66]. Among them, only one (SCB65560.1), to date, has been previously reported to be involved in virulence of *F*. *graminearum* [63]. The detection of positive selection in genes with protein/zinc ion binding domains, transcription factors and in genes encoding proteins involved in transmembrane transport (S1 Table) highlights their important role in driving evolutionary novelty that allow *F*. *graminearum* to increase adaptation to the host and/or environment. Transcription factors including Zn-binding proteins are well-known to control virulence of a range of pathogens and play an important role in microbial competition [76,77]. Zinc can either directly interact with virulence factors such as metalloproteases or Sods, or indirectly controls the expression of proteins essential for infection [78]. In addition, Zn-binding proteins seem to be crucial for phenotypic plasticity and adaptability of fungi through their involvement in a wide range of biological functions, such as metabolism, proteolysis, protein biosynthesis, transport, cell signaling, protein folding, transcription regulation, RNA processing, DNA replication/integration/repair, response to oxidative stress and antimicrobial resistance [76–80]. Besides transcription factors, several genes with signatures of selection were associated with membranes, transmembrane transport and metal ion transmembrane transport (S1 Table). Membrane transporters are involved in transport of a wide variety of substrates across extra- and intracellular membranes [81] and might play an important role in pathogenesis and stress response of pathogens [82,83]. Proteins associated with membrane functions are also involved in the development of tolerance to fungicides pointing towards efficient adaptation mechanisms to antifungal agents [84,85]. However, it should be underlined that national or regional differences in fungicide applications cannot explain the observed evolutionary findings, because both the EE and WE populations occupy the same zone of pesticide registration, where the same fungicidal mode of action groups are available [86].

Adaptive evolution, also known as positive selection, is a selective pressure exerted to a protein in response to an environmental change, which may lead to the increase of the population fitness in that environment [87]. Our results showed that, compared to the EE population, the WE population appears to be more likely under positive selection, as more outliers had unique variation consistent with a selective sweep of distinct loci/mutations (S1 Table, Fig 4). If populations differ in positive selective pressures, divergent selection may maintain different sets of alleles and result in increased differentiation [88]. Increased genetic diversity of *F*. *graminearum* may therefore provide a reservoir of genetic variation that can influence its adaptability. Analysis of accessory gene pool in *F*. *graminearum* showed that populations also maintained distinct sets of accessory genes, which, however, were more abundant in WE population. The WE population harbored accessory genes encoding proteins with functions related to oxidation-reduction, playing a crucial role in oxidative stress responses of various pathogens during infection process [89]. Proteins associated with processing of oxygen appear to play an important role during the invasion and colonization of host tissues, when exposed to oxygen-limited or hypoxic microenvironments during fungal pathogenesis [90].

These genes were absent in the EE accessory pool. The identified differences between two populations may suggest that they may follow different evolutionary trajectories likely reflecting unique virulence, pathogenicity and/or adaptation characteristics. The discovery of genes linked to these processes may support the above consideration. Indeed, we found that WE population harbored two proteins CAF3454557.1 and CAF3454395.1 showing high homology to tannase/feruloyl esterase (IPR011118), an enzyme known for its ability to degrade plant tannins; plant secondary metabolites characterized as plant defensive molecules [91]. Gitonga et al. (2022) [92] found that a long rain season led to increased production of tannins in cowpea, giving rise to the speculation that host plants in WE may have contained more tannins due to a more maritime climate in WE compared with EE, which in turn may have selected fungal strains with tannase-like proteins in WE, but not in EE. Further studies are needed to evaluate the functional significance of the revealed diversity in the epidemiology of *F*. *graminearum*. Verification of the functional variations and the molecular mechanisms associated with these allelic correlations may be critical to understand the complex phenomenon of pathogenicity in order to design novel and effective disease management strategies for controlling the disease. Future challenges to effective plant protection could arise from the dissemination of adaptations among populations resulting from introduction of new alleles through gene flow and recombination since selection may continue to promote the formation of highly adapted strains. Therefore, ongoing global surveillance will be essential in this regard for tracking population persistence and dynamics under changing climatic conditions. Finally, high-throughput surveillance of the emerging strains carrying diverse combinations of phenotypic and genotypic properties will be crucial for effective control of the pathogen.

## Supporting information

S1 Fig Population genetic structure of the estimated delta K value for seven run replication of each from K1 to K8. (EPS)

S2 Fig Population structure analysis of *F*. *graminearum* populations without outliers. (a) Principal component analysis (PCA) of a set of *F*. *graminearum* strains detecting three distinct populations: A1, East European (EE) and West European (WE). (b) A phylogenetic network constructed from a set of *F*. *graminearum* strains by reference-based mapping to the reference PH-1 strain. (c) Results of STRUCTURE analysis. Three different colors indicate clustering assignment of each isolate in the three populations inferred from Bayesian analyses: A1 = yellow, East European (EE) = green and West European (WE) = purple. Legend: Strains marked with red font indicate admixed strains. A1 includes seven strains of diverse geographical origin.
(EPS)

S3 Fig Phylogenies of European populations of *F*. *graminearum* with and without outliers. (a) A maximum-likelihood (ML) phylogeny inferred from SNP including outliers. (b) A maximum-likelihood (ML) phylogeny inferred from SNP excluding outliers. Legend: A maximum-likelihood (ML) phylogenies inferred from SNPs identified by reference-based mapping of whole genome sequences to the reference PH-1 strain (accession number GCF_000240135.3). Three different colors indicate clustering assignment of each isolate in the three populations inferred from Bayesian analyses: A1 = yellow, East European (EE) = green and West European (WE) = purple. Strains marked with red font indicate admixed strains. Six strains from FGSC were chosen as outgroup: *F*. *boothii* (CBS 110251 and CBS 119170), and *F*. *louisianense* (CBS 127524 and CBS 127525) and *F*. *gerlachii* (CBS 119175 and CBS 119176). The trees were rooted with *F*. *boothii* and drawn to scale, with branch lengths measured in the number of substitutions per site.
(EPS)

S1 Table. A List of strains used for Whole-Genome Sequencing (WGS) analyses and sequencing parameters. B. Determination of TRI genotypes based on sequence comparisons to refence alleles of NIV, 3-cADON, 15-AcDON and NX-2 genotypes. C. Genomic regions showing genetic signatures of selection in *F*. *graminearum* populations. D. Distribution and characteristics of genes differentially conserved among *F*. *graminearum* populations.
(XLSX)
